# Performance of Emergency Heart Failure Mortality Risk Grade in the Emergency Department

**DOI:** 10.5811/westjem.2021.1.48978

**Published:** 2021-04-08

**Authors:** Nidhi Garg, Renee Pekmezaris, Gerin Stevens, Adan Z. Becerra, Andrzej Kozikowski, Vidhi Patel, Ghania Haddad, Phillip Levy, Pridha Kumar, Lance Becker

**Affiliations:** * Northwell Health, Southside Hospital, Department of Emergency Medicine, Bayshore, New York; † Northwell Health, Department of Internal Medicine, Manhasset, New York; ‡ Northwell Health, Department of Cardiology, Manhasset, New York; § Rush University Medical Center, Department of Surgery, Chicago, Illinois; ¶ National Commission on Certification of Physicians Assistants, John’s Creek, Georgia; || Northwell Health, Department of Emergency Medicine, Manhasset, New York; # Wayne State University School of Medicine, Department of Emergency Medicine, Detroit, Michigan; ** Northwell Health, Long Island Jewish Medical Center, Department of Emergency Medicine, New Hyde Park, New York

## Abstract

**Introduction:**

The purpose of this study was to validate and assess the performance of the Emergency Heart Failure Mortality Risk Grade (EHMRG) to predict seven-day mortality in US patients presenting to the emergency department (ED) with acute congestive heart failure (CHF) exacerbation.

**Methods:**

We performed a retrospective chart review on patients presenting to the ED with acute CHF exacerbation between January 2014–January 2016 across eight EDs in New York. We identified patients using codes from the International Classification of Diseases, 9th and 10 Revisions, or who were diagnosed with CHF in the ED. Inclusion criteria were patients ≥ 18 years of age who presented to the ED for acute CHF. Exclusion criteria included the following: end-stage renal disease related heart failure; < 18 years of age; pregnancy; palliative care; renal failure; and “do not resuscitate” directive. The primary outcome was seven-day mortality. We used mixed-effects logistic regression models to estimate C-statistics and continuous net reclassification index for events and nonevents.

**Results:**

We identified 3,320 ED visits associated with suspected CHF among 2,495 unique patients. Of the 3,320 ED visits, 94.7% patients were admitted to the hospital and 3.4% were discharged. The median age was 78.6 (interquartile range 68.01 – 86.76). There was an overall seven-day mortality of 2%, an inpatient mortality rate of 2.4%, and no mortality among the discharge group. Adding EHMRG to the risk prediction model improved the C-statistic (from 0.748 to 0.772) and led to a higher degree of reclassification for both events and nonevents.

**Conclusion:**

The EHMRG can be used as a valuable and effective screening tool in the US while considering disposition decision for patients with acute CHF exacerbation. Emergency medical services transport and metolazone use is much higher in the US population as compared to the Canadian population. We observed minimal to no short-term mortality among discharged CHF patients from the ED.

## INTRODUCTION

### Background

Nearly 700,000 emergency department (ED) visits were due to acute heart failure (AHF) in 2009.^1^–^4^ Most visits result in a hospital admission and account for the largest proportion of the projected $70 billion that will be spent on heart failure care by 2030.^4^,^5^ There are few prognostic algorithms to guide in the decision to either admit or discharge a patient appropriately. Thus, clinicians may hospitalize some low-risk patients who have HF and may discharge home high-risk patients without being able to accurately assess prognosis.^6^ The lack of an accurate prognostic algorithm may be a contributing factor to the 80% admission rate for ED patients with AHF in the United States, which has remained unchanged over the last several years.^7^

A multicenter, Canadian cohort study reviewed the data of approximately 12,500 patients to derive and validate a risk model for predicting acute mortality in patients with HF who present to the ED. The randomly selected patients from 86 hospitals in the province of Ontario had visited an ED for HF and were discharged or hospitalized between April 1, 2004–March 31, 2007 (an average of 36 patients per hospital per year). Based on this data and assessing different variables such as age, transportation by emergency medical services (EMS), systolic blood pressure, heart rate, oxygen saturation (SpO_2_), creatinine, potassium, troponin, active cancer, and metolazone use at home, the researchers calculated an Emergency Heart Failure Mortality Risk Grade (EHMRG). The EHMRG served to stratify seven-day mortality risk after initial presentation with AHF, regardless of whether the patient was discharged from the ED or hospitalized.^8^

A recent study by Lee et al examined the validation of the EHMRG score compared to physician-estimated risk of seven-day mortality.^12^ Building on their conclusion that the EHMRG model proved to be a better indicator of seven-day mortality than physician estimates, we aimed to further validate this score by conducting a retrospective chart review to create a risk stratification of patients presenting to the ED with acute CHF exacerbation. There have been no studies performed in the US and independent of the original author to validate this score.

The purpose of our study was to validate and assess the performance of the EHMRG to risk stratify adult patients presenting to ED with acute CHF exacerbation based on seven-day mortality in our US population. Our secondary goal was to study the demographic patterns, disposition rates, and ED re-visit rate of CHF patients.

## METHODS

### Study Design and Setting

We conducted a retrospective chart review of patients presenting to the ED with acute CHF exacerbation between January 2014–January 2016. We extracted data from all patients admitted or discharged with an *International Classification of Diseases*, 9^th^ or 10^th^ Revision, (ICD 9 or 10) code for CHF as entered by an emergency physician. We obtained approval from our institutional review board before study initiation.

### Selection of Participants

We collected data from eight EDs across the largest health system in New York via the electronic health record (EHR) system (Allscripts Healthcare Solutions, Chicago, IL). Of the eight EDs, four were located at tertiary care centers and four were at community centers spanning the boroughs of Manhattan and Queens and the counties of Nassau and Suffolk. The total number of annual visits was 442,059 in 2014 and 473,387 in 2015 (see Appendix B for the detailed distribution of volume per hospital site). Inclusion criteria included adult patients at least 18 years of age or older who presented to the ED for acute CHF categorized using ICD codes for CHF. We excluded patients who were younger than 18 and those who presented to the ED with HF related to end-stage renal disease. We also excluded patients who were pregnant or had renal failure. Patients receiving palliative care and patients who had a “do not resuscitate” directive on file were also excluded from the study.

**Population Health Research Capsule**

What do we already know about this issue?

*The Emergency Heart Failure Mortality Risk Grade (EHMRG) has been validated in Canada to predict 7-day mortality but has not yet been validated in the United States (US).*

What was the research question?

*We assessed the performance of the EHMRG to predict 7-day mortality in patients presenting with congestive heart failure (CHF) exacerbation.*

What was the major finding of the study?

*The EHMRG adequately improved risk prediction of 7-day mortality for CHF-related ED visits in the US.*

How does this improve population health?

*Its use as a screening tool for the disposition decision of CHF-related ED visits encourages evaluation for social factors that may contribute to unsafe discharge.*

### Measurements

We collected both demographic and clinical data. Data abstraction was largely conducted electronically to reduce the error of documentation. Data not retrievable by electronic means, such as whether the patient was transported by EMS or used metolazone, was retrieved by three trained research associates who were blinded to the study hypothesis. The presence of active cancer was objective information obtained by chart extraction, and its clinical determination was made by chart review and confirmed with evidence of treatment. Troponin values were sorted as “yes” if values were above normal baseline levels and as “no” if values were not above normal levels (Table 1). Only the principal investigator and co-investigators had access to the data. The data was provided by a data analyst in a password-secured file and imported into REDCap (Research Electronic Data Capture, Vanderbilt University, Nashville, TN). Since EHMRG is a multivariable equation, an electronic, REDCap-based formula was developed to calculate the score and reduce the error of calculation. The data sheet was set to record the individual factors of EHMRG and then calculate the score. A pilot test was successfully conducted prior to the chart review.

### Outcomes

Our primary outcome was seven-day mortality in these patients. The mortality data was collected by a proxy system. For patients who were discharged before seven days from the ED visit, we reviewed their EHR for subsequent clinic visit or revisit to any point of contact in our health system. The patients were flagged as deceased by the insurance provider, and dates of death were recorded to calculate mortality.

### Analysis

We conducted descriptive analyses for all variables to assess their distribution. Categorical variables are presented as percentages and continuous variables as medians with interquartile ranges (IQR). All ED visits (including multiple ED visits for some patients) were used in multivariable analysis. We used two separate mixed-effects logistic regression models to allow for multiple ED visits per patient. We clustered by patient health record number in order to validate the assumption of correlated outcomes in the same patient. The first model used the individual metrics to derive the EHMRG score as individual-level predictors. The second model added the EHMRG score to the first model to predict seven-day mortality.

To determine appropriate sample size, we used events per variable ratio criteria. With 10 variables, an adequate number of events would be 100. We also estimated odds ratios and 95% confidence intervals (CI) for the EHMRG score quantiles from the second model. The apparent area under the receiver-operating characteristic (ROC) curve was estimated and plotted in a graph for the second model. We also estimated the C-statistic of each model and estimated the difference in C-statistic and *P* value for difference in C-statistic. The C-statistic is a unitless index denoting the probability that a randomly selected subject who experienced the outcome will have a higher predicted probability of having the outcome occur compared to a randomly selected subject who did not experience the event. It can also be interpreted as the rank correlation between predicted probabilities of the event occurring and the observed response. Finally, we estimated the continuous net reclassification index for events and nonevents separately. All analyses were conducted using R Statistical software (R Foundation for Statistical Computing, Vienna, Austria).

## RESULTS

### Characteristics of Study Subjects

Over the study period we used ICD codes to identify 3,782 ED visits that met the inclusion criteria. Of these, 462 were excluded because they did not meet the inclusion criteria or had incomplete data, leaving a final sample size of 3320 ED visits among 2,495 unique patients. Disposition data was missing from 189 visits; 123 visits were excluded because of renal failure; 34 had missing data on EMS transport; six were missing cancer information; three were missing metolazone information; one was missing initial SpO_2_; 22 were missing creatinine levels; 83 were missing potassium serum data; and one was missing heart rate data. We found no evidence that visits with missing data were different than visits with data (*P* = 0.51); thus, we analyzed only those with complete data. Of the 3,320 ED visits included in the analysis, 3,144 patients (94.7%) were admitted to the hospital and 113 (3.4%) were discharged from the ED. Nineteen patients (0.6%) left against medical advice; 22 (0.7%) were observed; 20 (0.6%) cases were transferred; and two (0.0%) passed away in the ED.

The median age among all ED visits was 78.9 years old with 1,607 (48.4%) females, and the median length of stay for those admitted to the hospital was seven days. The predominant racial category was White, constituting 55.4% of the ED visits. Table 1 presents the overall distribution of demographics and disposition of all ED visits. Table 2 presents median EHMRG scores by disposition type.

**Figure 1:**
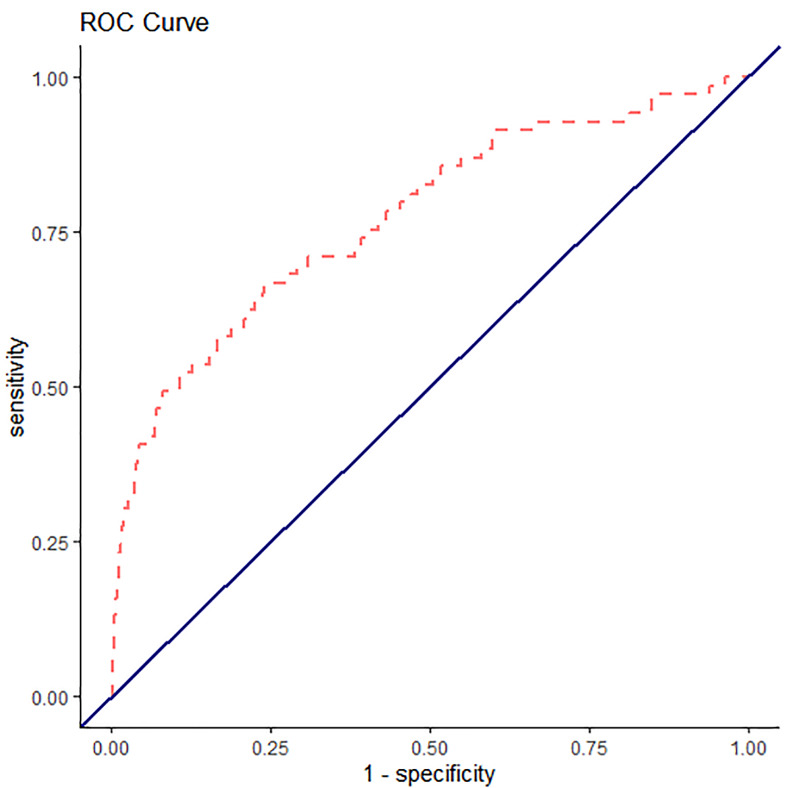
Receiver operating characteristic curve for seven-day mortality with the Emergency Heart Failure Mortality Risk Grade.

**Figure 2:**
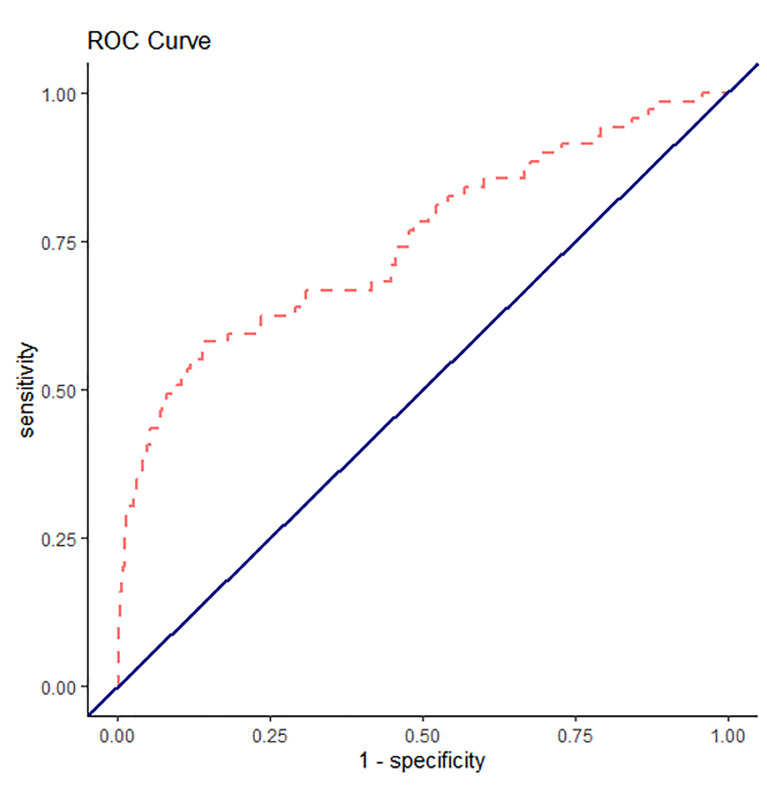
Receiver operating characteristic curve for seven-day mortality with individual covariates.

We fit two separate mixed-effects logistic regression models. The first logistic regression input all of the variables used to calculate EHMRG scores individually into the model. The second logistic regression model added the quantiles of the EHMRG scores. The C-statistic of the multivariable model using the individual variables was 0.748 (95% CI, 0.683 to 0.813) and the C-statistic of the model with EHMRG quantiles was 0.772 (95% CI, 0.729 to 0.815), suggesting that the model using EHMRG was superior for risk stratification (see Figures 1 and 2 for ROC curve). The C-statistic of the model with EHMRG was 0.024 (3.2% higher) than the C-statistic of the model with individual covariates, a significant improvement (*P* = 0.04). The net reclassification index for events was 0.25 and 0.13 for non-events. As shown in Table 3, patients who were in risk quantiles 5b had 6.16 times the odds of dying within seven days as compared to those in risk quantiles 1.

## DISCUSSION

In this study of CHF-related ED visits in a large US health system, we found that the EHMRG score adequately improved risk prediction of seven-day mortality, validating previous studies in other countries. Specifically, the C-statistic was higher when using EHMR quantile and this model led to 25% and 13% of events and non-events to be classified into a better risk category, respectively. Given that both the C-statistic and the net reclassification improvement both have limitations, we used both to produce consistent and robust results.

Our median age was 79, which is comparable to the median age of 75 in the original EHMRG study and supports the fact that CHF is an advanced and chronic illness, mostly affecting the elderly. In our study we found that more than half of ED visits (53%) used EMS as a transport to the ED, which was much higher than the original study that recorded 38.5–43.4%.^8^ We also found that metolazone was used twice as often in the Canadian data set; however, we had a lower percentage of patients with active cancer who were suffering from CHF exacerbation in our data set. Our mortality rate overall was very similar to the Canadian study. Despite having the same seven-day mortality rate in our population as compared to the Canadian study, we identified that hospitalization mortality rates were higher among patients who were admitted to the hospital, as one may expect. We had a much higher percentage of non-normal troponin levels, 24% as compared to 10.5–14.6% in the original data set. The creatinine levels, systolic blood pressure, heart rate, and mean potassium levels were similar in our data set as compared to the Canadian data set. We found a similar mean SpO_2_ level in our data set of 91.5% vs 92.9–93.9% in the original study.^8^

The Canadian study enrolled 12,500 patients from 86 hospitals over a three-year period, which amounts to an average of 36 patients per year per hospital. Patients were randomly selected. This was different from our selection of patients. However, the number of hospitals covered was greater and it adjusts for more variation and practice patterns.^8^ We had an admission rate of 94.7%, which was higher than the national average admission rate of 80–85%; we had 0 outpatient discharge seven-day mortality. Additionally, the ED revisit rate was 24.8%, which is the national US average for CHF patients.^9^

The US and Canadian healthcare systems are different and hence the risk involved in discharging a patient in the US is higher medically and legally, which would explain no mortality in the discharge group. However, in our system we still had a 24.8% ED revisit for CHF, which is similar to the national average. Therefore, it is unclear whether prior admission to the hospital prevents further revisits to the ED and hence readmissions for the same complaint. It is questionable whether the presence of a prior admission is helpful in decreasing the chance of a revisit to ED.^10^

The percentage of health costs paid by the government in Canada is 71.3% vs 49.1% in the US.^11^ There was no difference in the mortality rate of our data set as compared to the Canadian study. This leads us to explore the role of hospitalization on short-term mortality. It has been well established that hospitalization is an independent predictor of long-term mortality in CHF patients.

## LIMITATIONS

This was a retrospective study of a single (albeit large) health system. This hospital system is in the Northeast US, and the population is more diverse as compared to the rest of the country. All the patients were not unique in our study; however, we considered each CHF visit to be unique when a patient presented to the ED.

The predictive ability of the EHMRG score in our data set was less than the Canadian study. This may be due to the low power of the study (only 69 events). All patients in our data set who had a seven-day mortality were hospitalized, which leads us to question the impact of hospitalization on mortality. A lack of cohort of discharged patients with seven-day mortality is a significant limitation of our study, as it depicts that the US population and culture of medicine are quite different than those of Canada. Another limitation was the use of ICD codes to identify patients with CHF (as opposed to Framingham heart failure diagnostic criteria), as it was practically difficult to apply the criteria retrospectively due to documentation limitations.^8^ Finally, we did not account for patients who were diagnosed with CHF exacerbation *after* admission to the hospital.

## CONCLUSION

In summary, risk prediction of seven-day mortality was superior when using a model that implemented the Emergency Heart Failure Mortality Risk Grade compared to a model that used the individual components of the EHMRG as covariates. EHMRG can be used as a good screening tool in the US while considering the disposition decision for these patients presenting with acute exacerbation of CHF. The objective evaluation may point physicians to evaluate for social factors that might contribute to unsafe discharge or poor outcomes. Potentially, some of the low-risk patients can be evaluated for social needs by a case manager or social worker, if available in the ED, and be safely discharged. This will potentially avoid some admissions and hence readmissions for the same issue.

## Supplementary Information



## Figures and Tables

**Table 1:** Patient characteristics, overall and stratified by mortality.

Characteristics	Overall (N = 3320) n (%)	Died within 7 days (N = 69, 2%) n (%)	Alive (N = 3251 98%) n (%)
Age (median [Q1–Q3])	78.7 (68.13–86.7)	86.3 (77.3–90.82)	78.6 (68.0–86.5)
Gender			
Male	1,710 (51.5)	27 (1.6)	1,683 (98.4)
Female	1607 (48.4)	42 (2.6)	1,585 (97.4)
Unspecified	3 (0.1)	0 (0)	3 (100)
Race/ethnicity			
White	1,838 (55.4)	44 (2.4)	1,794 (97.6)
Black	838 (25.2)	11 (1.3)	827 (98.7)
Asian	211 (6.4)	4 (1.9)	207 (98.1)
Other	337 (10.2)	3 (0.9)	334 (99.1)
Declined	13 (0.4)	0 (0)	13 (100.0)
Unknown	73 (2.2)	7 (9.6)	66 (90.4)
Troponin	258 (1.9)	57 (2.8)	25 (1.9)
Upper limit of normal	811 (24.4)	33 (4.1)	778 (95.9)
Normal	2,509 (75.6)	36 (1.4)	2,473 (98.6)
EMS transport			
Yes	1,742 (52.5)	54 (3.1)	1,688 (96.9)
No	1,578 (47.5)	15 (1.0)	1,563 (99.0)
Active cancer			
Yes	163 (4.9)	8 (4.9)	155 (95.1)
No	3,157 (95.1)	61 (1.9)	3,096 (98.1)
Metolazone			
Yes	132 (4.0)	1 (0.8)	131 (99.2)
No	3,188 (96.0)	68 (2.1)	3,120 (97.9)
Disposition			
Admitted	3,144 (94.7)	67 (2.1)	3,077 (97.9)
AMA	19 (0.6)	0 (0)	19 (100)
Discharge	113 (3.4)	0 (0)	113 (100)
Observation	22 (0.7)	0 (0)	22 (100)
Transfer	20 (0.6)	0 (0)	20 (100)
Expire	2 (0.0)	2 (100)	0 (0)
Systolic blood pressure (mm Hg; median [Q1–Q3]*)	139.0 (121.0–159.0)	122 (104.0–146.0)	139.0 (121.0–159.0)
Heart rate (beats/min; median [Q1–Q3]*)	82.0 (70.0–96.0)	83.0 (72.0–98.0)	82.0 (70.0–96.0)
SpO_2_ (%; median [Q1–Q3]*)	97.0 (95.0–99.0)	96.0 (94.0–99.0)	97.0 (95.0–99.0)
Creatinine (mg; median [Q1–Q3]*)	1.3 (1.0–1.9)	1.5 (1.1–2.2)	1.3 (1.0–1.9)
Potassium (mg; median [Q1–Q3]*)	4.3 (3.9–4.8)	4.5 (4.0–5.1)	4.3 (3.9–4.8)
EHMRG score (median [Q1–Q3]*)	24.7 (−19.9–71.1)	103.5 (32.3–166.9)	23.6 (−20.6–69.7)

Main Results:

* Q1: First Quartile. Q2: Third quartile. Sixty-nine (2.0%) of the ED visits led to death within 7 days of ED presentation, while 3,251 (98.0%) survived 7 days from discharge. No patients died within 7 days of discharge from ED in our study population. The median EHMRG score among the sample was 24.7 (Q1–Q3 = 19.9–71.1).

*EMS*, emergency medical services; *AMA*, against medical advice; *SpO_2_*, oxygen saturation; *EHMRG*, Emergency Heart Failure Mortality Risk Grade; *mm Hg*, millimeters mercury; *mg*, milligram; *ED*, emergency department.

**Table 2:** Mean Emergency Heart Failure Mortality Risk Grade scores by disposition type.

	Admitted	AMA	Discharged	Observation	Transferred	Expired in ED
EHMRG score	26.5	15.4	−16.1	−35.5	44.7	43.3

*EHMRG*, Emergency Heart Failure Mortality Risk Grade; *AMA*, against medical advice; *ED*, emergency department.

**Table 3:** Results of mixed-effects logistic regression model for 7-day mortality (n = 3,320).

Risk quantiles	Score range	7-day mortality rate %	OR	95% CI	P-value
1	≤ −49.1	0.91		Reference	
2	−49.0 to −15.9	1.11	1.22	0.33 – 4.58	0.77
3	−15.8 to 17.9	0.63	0.69	0.17 – 2.78	0.60
4	18.0 to 56.5	1.50	1.65	0.52 – 5.21	0.39
5a	46.6 to 89.3	1.56	1.72	0.50– 5.91	0.39
5b	≥ 89.4	6.16	7.12	2.52 – 20.09	<0.001

*OR*, odds ratio; *CI*, confidence interval.
