# Supplementary material for: Performance of Emergency Heart Failure Mortality Risk Grade in the Emergency Department
Source: West J Emerg Med. 2021 Apr 8;22(3):672–7. doi: 10.5811/westjem.2021.1.48978 (PMC8203016; doi:10.5811/westjem.2021.1.48978)
Supplement: Supplementary file 1 [file wjem-22-672-s001.pdf]

Appendix A. Emergency Heart Failure Mortality Risk Grade (EHMRG)

Variable	Units	Additive or Multiplicative Component
Age	y	2 × age
Transported by EMS	If "yes"	+60
SBP	mm Hg*	−1 × SBP
Heart rate	beats/min†	1 × heart rate
Oxygen saturation	%‡	−2 × oxygen saturation
Creatinine	mg/dL§	20 × creatinine
Potassium	4.0 to 4.5 mmol/L	0
	≥4.6 mmol/L	+30
	≤3.9 mmol/L	+5
Troponin	>ULN	+60
Active cancer	If "yes"	+45
Metolazone at home	If "yes"	+60
Adjustment factor‖		+12
**Total**		**EHMRG score¶**

EHMRG = Emergency Heart Failure Mortality Risk Grade; EMS = emergency medical services; SBP = systolic blood pressure; ULN = upper limit of normal.
* Initial/triage SBP, maximum of 160 mm Hg.
† Initial/triage heart rate, minimum of 80 beats/min and maximum of 120 beats/min.
‡ Lowest initial/triage oxygen saturation, maximum of 92%.
§ If creatinine concentration is in μmol/L, divide by 88.4 to convert to mg/dL.
‖ Adjustment factor of +12 added to allow for an approximate 0 median score.
¶ All variables are required to calculate the score; users are cautioned against estimating component values. The EHMRG is not for use in patients who are dialysis-dependent.

Appendix B

Site	2014	2015	2016
LIJ	88,258	94,003	97,735
SS	71,711	70,061	69,134
FH	51,750	54,330	56,530
PV	33,356	33,459	33,557
VS	42,210	43,561	42,765
LHGV*	11,826	33,666	37,274
NSUH	87,511	89,066	90,035
LHH	55,437	55,241	54,423

* LHGV opened 7/1/14
